# Evaluation of Metabolomics as Diagnostic Targets in Oral Squamous Cell Carcinoma: A Systematic Review

**DOI:** 10.3390/metabo13080890

**Published:** 2023-07-27

**Authors:** Susanth Alapati, Giulio Fortuna, Gordon Ramage, Christopher Delaney

**Affiliations:** 1 Oral Sciences Research Group, Glasgow Dental School, School of Medicine, Dentistry and Nursing, University of Glasgow, 378 Sauchiehall Street, Glasgow G2 3JZ, UK; susanth.alapati@abdn.ac.uk (S.A.);; 2 Department of Oral Medicine, Glasgow Dental School, School of Medicine, Dentistry and Nursing, University of Glasgow, 378 Sauchiehall Street, Glasgow G2 3JZ, UK

**Keywords:** OSCC, metabolomics, metabolites, oral cancer, biomarkers, cancer diagnosis

## Abstract

In recent years, high-throughput technologies have facilitated the widespread use of metabolomics to identify biomarkers and targets for oral squamous cell carcinoma (OSCC). As a result, the primary goal of this systematic review is to identify and evaluate metabolite biomarkers and their pathways for OSCC that featured consistently across studies despite methodological variations. Six electronic databases (Medline, Cochrane, Web of Science, CINAHL, ProQuest, and Embase) were reviewed for the longitudinal studies involving OSCC patients and metabolic marker analysis (in accordance with PRISMA 2020). The studies included ranged from the inception of metabolomics in OSCC (i.e., 1 January 2007) to 30 April 2023. The included studies were then assessed for their quality using the modified version of NIH quality assessment tool and QUADOMICS. Thirteen studies were included after screening 2285 studies. The majority of the studies were from South Asian regions, and metabolites were most frequently derived from saliva. Amino acids accounted for more than quarter of the detected metabolites, with glutamate and methionine being the most prominent. The top dysregulated metabolites indicated dysregulation of six significantly enriched pathways including aminoacyl-tRNA biosynthesis, glutathione metabolism and arginine biosynthesis with the false discovery rate (FDR) <0.05. Finally, this review highlights the potential of metabolomics for early diagnosis and therapeutic targeting of OSCC. However, larger studies and standardized protocols are needed to validate these findings and make them a clinical reality.

## 1. Introduction

Oral cancer is one of the most prevalent cancers in the head and neck region [1]. It is the 6th most common cancer globally, with an incidence of around 377,713 new cases reported in the year 2020 and a projected increase of over 40% by 2040 [2]. The most surprising aspect of oral cancer is that, despite its development associated with modifiable risk factors, and the oral cavity being the most accessible region for examination, it still poses a challenge to the healthcare system with a 5-year survival rate of 20–40% [3,4,5,6]. The survival rate is due to the majority of oral cancer lesions being either undetectable in earlier stages or only being presented to the clinician in advanced stages. Clinical studies have shown that the survival rate of this malignancy can be improved to greater than 80%, if detected in stage 0 (carcinoma in situ) or even stage I [7,8]. Therefore, a reliable and trustworthy diagnostic/adjunctive diagnostic tool such as molecular biomarkers are required to predict oral malignancy in the precancer/earlier stage with the potential to improve the treatment outcome and prognosis.

In recent years, it is well established that chromosomal instability at the genetic level, exhibited as alterations or mutations, drives multistep carcinogenesis [9]. Furthermore, oral mucosal damage is established at the genetic or epigenetic level long before it is manifested clinically or histologically [10]. As a result, addressing these molecular pathways and discerning the mechanisms underpinning the development of oral cancer might provide a strategy for mitigating the mortality and morbidity caused due to oral cancer. This argument has prompted extensive research to identify novel, sensitive and specific biomolecules that may serve as a less-invasive diagnostic tool in predicting the risk of malignancy before clinical and histological alterations occur [11]. 

In fact, recent technological advances have assisted in better identifying and characterising tumour biomarkers. One such biomedical advancement is the discipline of “OMICS”, which comprises various thrust areas such as genomics, proteomics, transcriptomics, and metabolomics [12]. For the past two-and-a-half decades, 75% of the research related to this utilised either genomic or proteomic techniques, revolutionising decision-making in targeted drug therapy and personalised medicine in cancer [13,14]. However, although genomic and proteomic studies provide a thorough understanding of genotype and expressed phenotype, they do not fully represent the altering phenotype. Hence, it might be preferable to examine the downstream changes occurring at metabolite levels to potentially understand oral cancer better [15,16]. Metabolomics is one such rapidly developing high-throughput method that is often used to identify the metabolites and quantify their concentration present in a given sample at a snapshot of time [17,18,19].

The clinical literature on metabolomics and oral cancer is growing and the field of metabolomics-based oral cancer research is a novel, complex, dynamic and moving landscape [17,18,19]. In recent years, the interest in oral cancer metabolomics is increasing and has been applied in various dimensions of its research. Individual studies have identified a panel of metabolite biomarkers that not only discriminated between OSCC from healthy controls/OPMDs but also potentially paved the way to better understanding the pathophysiology of OSCC and tackling the huge challenge of its early-stage diagnosis [11,17,20,21,22,23].

However, most of the studies regarding OSCC metabolomics have smaller sample sizes, which makes it difficult to validate their findings and establish generalisability across various populations. Moreover, a growing number of authors over the last decade have subsequently incorporated this evidence on the OSCC metabolomics as a descriptive and subjective overview of the literature. However, there is a dearth of studies that reported OSCC metabolomics in a systematic review approach. As a result, the overarching goal of this study is to systematically identify and evaluate metabolite biomarkers for OSCC that featured consistently across various metabolomic studies despite methodological heterogeneity. This would ensure a comprehensive and adequate coverage of metabolic biomarkers across a variety of study designs and encourage further oral cancer research into better understanding the pathophysiological alterations occurring at molecular level and identifying new metabolite biomarkers and a specific set of pathways.

## 2. Materials and Methods

This research was designed a priori in accordance with the updated guidelines for systematic review provided by the PRISMA 2020 statement [24]. The detailed protocol was registered on PROSPERO (CRD42022373177).

### 2.1. Research Question

The following focussed research question was formulated using PECOS structured framework [25]. 

“Are there any common dysregulated metabolites (O) that distinguish adult patients (P) with a confirmed diagnosis of OSCC (E) and healthy patients or OPMDs (C) regardless of the methodological heterogeneity across various metabolomic studies, which can potentially aid in early diagnosis and prognosis of OSCC?”.

### 2.2. Search Strategy

A comprehensive and unique approach was implemented to search six electronic databases, including Medline (Ovid), Embase, EBM Reviews, CINAHL, Web of Science and ProQuest. To search each of these databases, a unique search strategy based on the combination of keywords paired with the Boolean operators “AND” and “OR” were utilised (Supplementary Table S1). The search was conducted since the early adoption of metabolomics in oral cancer, i.e., 2007 until 30 April 2023. The studies were collected and stored in the Mendeley reference manager software version 1.19, followed by the identification of duplicates using the same software [26]. Two independent authors implemented the complete search method. 

### 2.3. Study Inclusion and Exclusion Criteria

The detailed study selection criteria were presented according to the PECOS domains [25] as outlined below:

#### 2.3.1. Inclusion Criteria

- P (Participants): Adult patients (>18 years of age) from any geographic location, any age or gender.
- E (Exposure): Patients with confirmed diagnosis of OSCC.
- C (Comparison): Difference in concentration of metabolites between OSCC and predetermined controls.
- O (Outcomes): Dysregulation of metabolite concentrations between the predetermined study groups, which are reported as either mean ± standard deviation, fold change concentration or log fold change concentration.
- S (Study Design): Human-based observational studies (case-control, cohort, or cross-sectional) published since inception of OSCC metabolomics, i.e., January 2007 and April 2023 that used a metabolomic technique to quantify metabolite concentration.

#### 2.3.2. Exclusion Criteria

- Patients diagnosed with neoplasms other than OSCC either in the past or currently.
- Patients suffering from any reported chronic systemic illness or on medication for the same.
- Patients with oral lesions due to associated dermatological diseases, infections, localised trauma, recurrent aphthous ulcers, and systemic conditions.
- Targeted metabolomic experiments that are used to validate and translate already identified metabolites from hypothesis generating studies.
- Components other than metabolites as biomarkers such as genetic and protein.
- Animal or cell-based studies.
- Non-observational study designs such as case reports, conference proceedings, letters to editor, reviews, and meta-analysis.
- Metabolites quantified other than in concentration such as field of appearance, retention time, *m*/*z* ratio, etc.
- Studies published before January 2007 or after April 2023.

### 2.4. Study Selection

Following the application of inclusion and exclusion criteria, study selection was carried out in two stages by two independent reviewers using RAYYAN AI Systematic Review automation tool version 1.1.0 [27]. The first stage includes screening titles and abstracts in all electronic databases. Studies that matched the inclusion or had insufficient data in abstracts were chosen for the next phase. In the next phase, the complete text of the included studies from the initial phase was assessed to ascertain whether they met all eligibility criteria. A snowballing of all included studies was utilised to assess any references that were mistakenly omitted critically. During the entire study selection process, when a consensus could not be reached, a third independent reviewer was consulted to make a final decision. The complete text for each of the studies was used to make the final decision.

### 2.5. Data Extraction and Outcomes

Following study inclusion, the necessary information from the selected studies were extracted by two independent reviewers and then tabulated. The key results that were collected from the included studies were title, year published, country, author(s), sample size (cases & controls), diagnosis, comparison (case vs. control), type of sample, sample collection, handling, storage methods, metabolomic approach (targeted or untargeted), metabolites detected, metabolite names standardised in MetaboAnalyst 5.0 [28] and their concentrations (either as mean ± standard deviation, fold change concentration or log fold change concentration), analytical methods used, and key results reported in the respective studies. For those who reported metabolite concentrations as mean ± standard deviation, fold change is calculated as case/control followed by logarithmic transformation.

Primary outcomes of the included studies were fold-change concentrations of the metabolites between case and control (and/or) mean ± standard deviation concentration. Following the identification of metabolites, the MetaboAnalyst 5.0 online platform was used to identify these metabolites in databases such as The Human Metabolome Database (HMDB) [29], Kyoto Encyclopaedia of Genes and Genomes (KEGG) [30], PubChem [31] and Lipid maps [32]. Unidentified metabolites were not archived.

### 2.6. Data Synthesis

Given the heterogeneity of sample type, their collection, handling, storage and analytical methods used to evaluate their concentrations and limited availability of patient specific raw data, it was pertinent to perform a systematic review without pooling data for metanalysis following PRISMA and SWiM reporting guidelines. Hence, the included studies and their results are presented in a systematic narrative format, beginning with an overview of the quality assessment of the included studies and ending with a detailed description of metabolites with a focus on the most featured metabolites and their dysregulated pathways.

### 2.7. Risk of Bias Assessment

In our study, the risk of bias and quality assessment of the included studies were assessed by two reviewers and any disagreements were resolved through discussion. This was evaluated using a modified version of the quality assessment tool for observational cohort and cross-sectional studies by the NIH [33] and QUADOMICS tool [34], which is specific to OMICS-based studies. To assess the quality of studies included in this systematic review, we addressed 12 selected questions for each to assess methodological subheadings used in metabolomic investigations. Each question is either answered ‘yes’, ‘no’ or ‘not clear’. This tool is used only to assess a study’s internal quality but not to determine individual study quality, as the cut-off for assessing individual study quality has not been published by NIH or QUADOMICS.

### 2.8. Statistical Analysis

The included studies were pooled together with the primary outcomes such as metabolite name, comparison order, log_2_ fold change in concentration or mean ± standard deviation. The pooled data were then analysed for their differentially regulated metabolites using R and visualised using ggplot2 in R-Studio and GraphPad Prism version 9. Furthermore, the metabolite biomarkers that featured in at least two studies were analysed for their pathway analysis using MetaboAnalyst 5.0. The hypergeometric enrichment method and topology analysis based on relative-betweenness centrality were then performed using the standard reference metabolome and KEGG pathway library. For a pathway to be identified as significantly enriched, an adjusted *p*-value (false discovery rate, FDR) of less than 0.05 was considered necessary.

## 3. Results

### 3.1. Search Strategy and Study Selection

**Figure 1:**
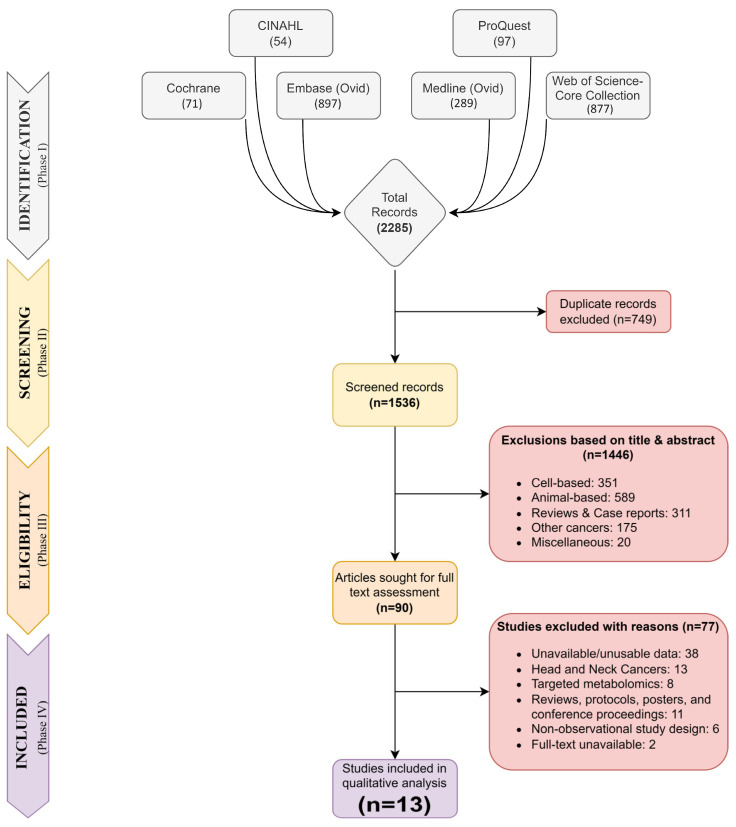
PRISMA flow diagram of search strategy and inclusion according to the PRISMA 2020 updated guidelines.

A total of 2285 records were retrieved after the identification phase, out of which 749 duplicate records were excluded from the initial screening. The remainder of the 1536 records were screened for their title and abstract, from which 1446 records were excluded from the full-text assessment. Ninety records were sought for phase IV screening, out of which 77 were excluded from inclusion because of their study design, targeted approach, un-usability in data or unavailability of the full text. Finally, 13 studies were included in the qualitative analysis. Because two of the studies included in this review had the same group of participants, we used different sample groups from each study to avoid any potential bias and ensure a wide range of metabolites [35,36]. Figure 1 depicts the flowchart summarising the study identification, screening, eligibility and inclusion process.

### 3.2. Study Characteristics

The characteristics of the 13 included studies are presented in Table 1. In brief, the selected studies were published in the last seven years (2016–2022) and were conducted largely (92%) in South Asian countries such as China, Japan, and India, followed by Brazil. Of the studies that reported age, sex and cancer staging, the OSCC group ranged in age from 23 to 94 years, with 56% of them being male. The OSCC cohort was further evenly distributed across all clinical cancer stages. Most of the research compared metabolomics data between OSCC and healthy controls, while only a few studies compared OSCC and OPMD to healthy controls. The bulk of the studies looked at metabolomic data in saliva followed by serum, tissues and urine. Mass spectrometer (MS) based technologies were the most widely employed analytical tools for quantifying the global metabolite concentration in the samples. Finally, in terms of study design, all the studies included used a case-control approach.

### 3.3. Risk of Bias and Quality Assessment

**Figure 2:**
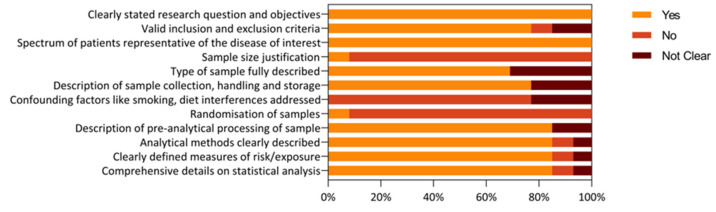
Summary of quality assessment of included studies based on the modified version of NIH quality assessment tool and QUADOMICS. The proportion of studies satisfying the criteria are plotted on a scale of 100%.

Secondly, we assessed the risk of bias and quality assessment findings obtained using the modified version of the QUADOMICS tool. According to the quality assessment, all of the included studies clearly outlined their research question and objectives with each, and every sample representative of the disease researched. While only a minority of the studies justified sample size (7.5%) and randomised the samples (7.5%), the bulk of them detailed the type of sample (70%), its collection, handling, and storage (85%), pre-analytical processing (85%), and the method used to quantify metabolites concentration (85%). Finally, just under 90% of the samples included a detailed description of the statistical methodology used to analyse the data shown in Figure 2 and Supplementary Table S2. 

### 3.4. Identification of Unique Metabolites

**Figure 3:**
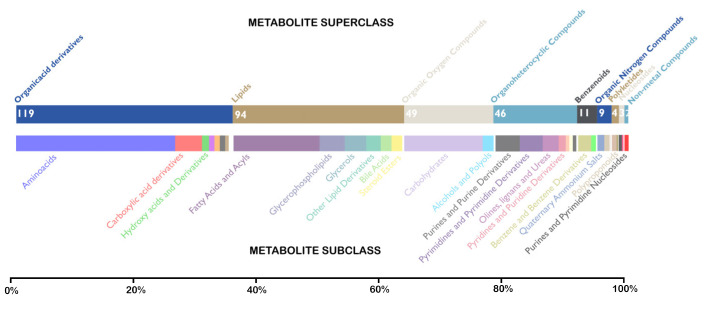
Stacked bar graph depicting the frequency of super-classes and sub-classes of distinct metabolites in various studies that reported metabolomics data of oral cancer (OSCC). The outer scale determines the percentage of metabolites, whereas inner numeric signifies the frequency. Different colours signify different metabolite super-classes and sub-classes.

A total of 337 distinct metabolites were identified from the studies included. Among these unique metabolites, 321 metabolites were differentially abundant between the OSCC and healthy controls, and 21 metabolites classified OSCC from OPMD. One-third of the obtained metabolites are organic acid derivatives (35%), followed by lipids (27%) and organic oxygen compounds (15%) on clustering into their super classes. On further clustering into subclasses, amino acids form the majority followed by fatty acids and carbohydrates. Figure 3 depicts the frequency of metabolite super classes and subclasses across 13 studies. The frequency of metabolite superclass and subclass is detailed in Supplementary Table S3.

### 3.5. Identification of Differentially Regulated Metabolites

**Figure 4:**
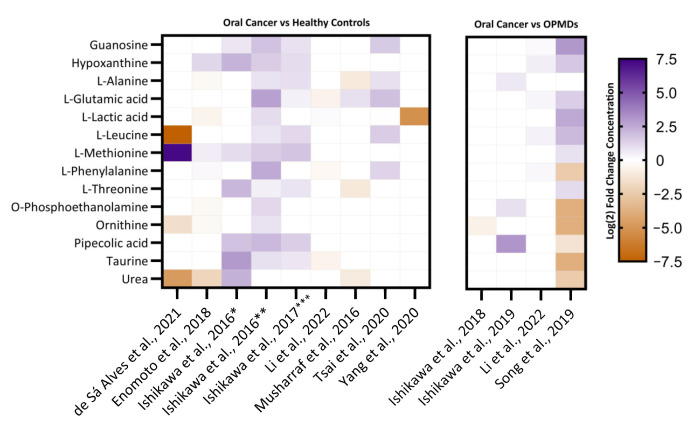
Heatmap depicting the comparison of log_2_ fold change of top featured metabolites in OSCC and OPMD across different studies and their samples [20,22,23,35,36,37,38,39,40,41,42,43,44]. The heatmap is faceted into two groups according to the comparison. Each row signifies log_2_ fold change of metabolite and are displayed as colours ranging from blue (highest concentration) to brown (least concentration) as shown in the legend, whereas each column signifies the study in which they are featured. The unfeatured metabolites are valued at ‘0’. The legend signifies the log_2_ fold change concentration of various metabolites. * Saliva, ** Tissue, *** Healthy vs. OC group 3.

**Figure 5:**
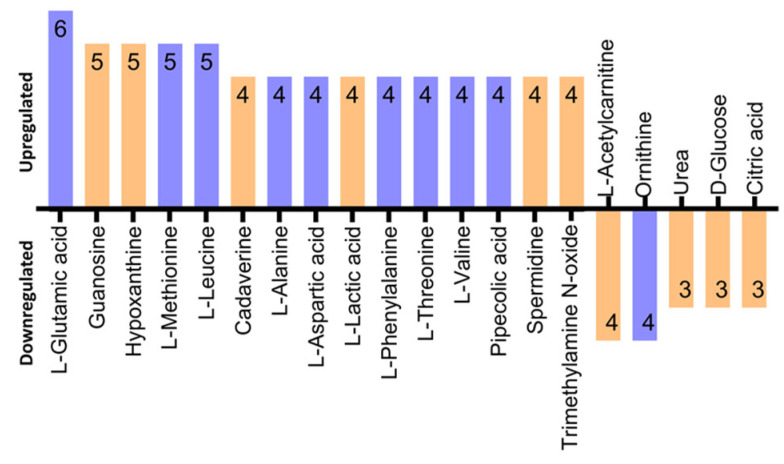
Bar graph depicting the frequency of top upregulated and downregulated metabolites in 13 studies that reported metabolomics data of oral cancer (OSCC). L-glutamic acid was the top upregulated metabolite which had six hits out of 13 studies. Blue coloured bar signifies metabolites of amino acids and their analogues, whereas the yellow coloured bar relates to non-amino acid classes.

Next, out of the deduced 337 unique metabolites, 14 differentially regulated metabolites were identified to be cited in at least five studies. On plotting the log_2_ fold change concentration of these metabolites as shown in Figure 4, we found that compared to healthy controls/OPMDs, most of the metabolites were upregulated in OSCC. An amino acid L-glutamic acid is the most featured metabolite, which appeared across six out of 13 included studies [22,23,35,36,42,43]. Glutamine was followed by another amino acids, L-methionine [22,35,36,37,38] and L-leucine [22,23,35,36,43] both of which were found to be upregulated in five studies respectively. Likewise, purine derivatives hypoxanthine [22,23,35,36,38] and guanosine [22,23,35,36,43] reported to be upregulated in five studies. Moreover, on further classifying the metabolites downregulated, we found that L-acetyl carnitine and Ornithine [22,37,38,42] are the two most downregulated metabolites reported in four studies (Figure 5).

### 3.6. Pathway Analysis of Top Featured Metabolites

**Figure 6:**
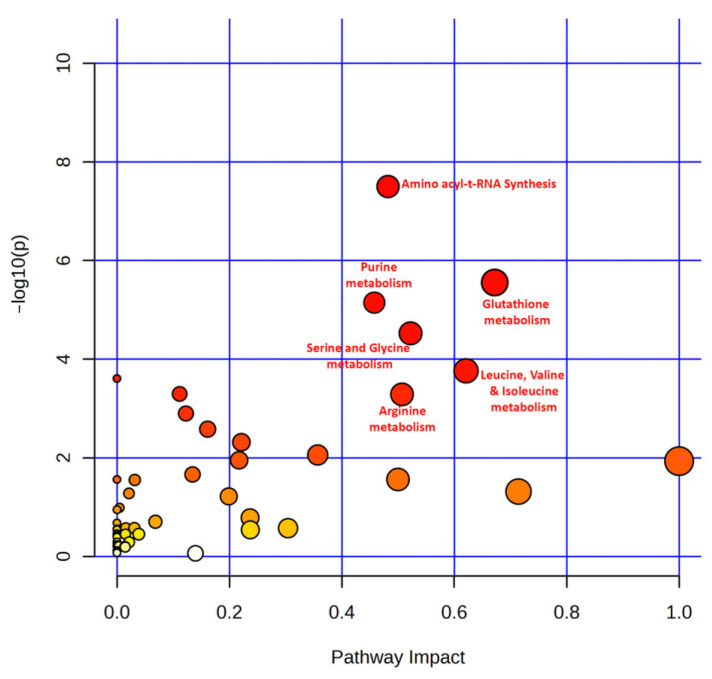
A scatter bubble graph illustrating the pathway analysis based on hypergeometric enrichment method of top 100 significantly dysregulated metabolites in OSCC. The *y*-axis depicts the log10 of *p*-value, while the *x*-axis represents the pathway impact values evaluated from the topology analysis; the circular node colour is determined by its *p*-value with red being most significant, and circular node size is determined by the pathway impact values. The most significantly altered pathways (FDR < 0.05) have a high log-(*p*) value as well as a high impact value (top right region).

Finally, we extracted 100 distinct metabolites from the included studies that were found to be dysregulated in more than one study. These metabolite candidates were submitted to the MetaboAnalyst 5.0 for pathway enrichment analysis. As shown in Figure 6, the pathway enrichment analysis demonstrated six dysregulated metabolic pathways with FDR < 0.05, including aminoacyl-tRNA biosynthesis (17/48 hits, Impact 0.49), glutathione metabolism (6/28 hits, Impact 0.67), arginine metabolism (8/14 hits, Impact 0.51), leucine, isoleucine and valine metabolism (4/8 hits, Impact 0.62), purine metabolism (10/65 hits, Impact 0.46), and serine and glycine metabolism (7/33 hits, Impact 0.53). Further information on the extracted metabolites and their enriched pathways can be found in the Supplementary Table S3.

## 4. Discussion

The central objective of this systematic review was to identify possible metabolite candidate biomarkers and their dysregulated pathways from various metabolomic studies on OSCC. Hence, in this systematic review, we screened 1536 research publications and identified 13 of them that met our selection criteria for quantitative analysis.

In general, the majority of the research on oral cancer metabolomics originated from the South Asian region, which accounts for more than 2/3rd of the OSCC case load worldwide [2]. This is because of the distinct cultural behaviours such as betel nut chewing, as well as varying patterns of alcohol and tobacco usage, both of which are designated as substantial risk factors for development of OSCC [45]. Saliva has been the sample of choice, followed by sera and tissue to capture the metabolomic picture of OSCC. This can be accredited to saliva’s ease of collection and proximity to the OSCC lesions [46,47]. Furthermore, compared to NMR, MS based (GC-MS, LC-MS & CPSI-MS) analytical technologies are most employed, owing to their low sample volume and potential to detect a broad range of metabolites. This also led us to observe no observable differences between the metabolites identified using these techniques. As the majority of the studies included did not offer raw data, rather they reported the fold change or mean and standard deviation of statistically significant metabolites, we integrated and analysed the latter.

### 4.1. Identification of Metabolite Candidate Biomarkers

The pooled data revealed 337 distinct metabolites. Lack of methodological homogeneity resulted in such a broad range of metabolites. One-third of the metabolites identified belong to the class of amino acids, the building block of proteins. This can be attributed to greater need for protein synthesis, owing to rapid cellular proliferation [48,49]. L-glutamic acid also known as glutamate (6/13 hits) followed by L-methionine (5/13 hits) were the most reported metabolites and amino acids across different studies. glutamate is known to be the primary nutrient source of tumours, which replenishes the TCA cycle via alpha ketoglutarate to synthesise citrate and fatty acids [50]. Furthermore, the nutritionally and oxygen deprived tumour microenvironment frequently relies on glutamine catabolism for energy and protein synthesis. Glutamate, the initial product of glutamine catabolism catalysed by glutaminase enzyme fuels the glucose-independent TCA cycle in a nutrient deprived tumour microenvironment [51]. Moreover, multiple studies have confirmed the enzyme glutaminase which converts glutamine to glutamate is over expressed in OSCC for their increased energy and proliferation needs and that its expression is correlated to poor prognosis in OSCC patients [52,53,54,55]. Next, considering L-Methionine, the primary methyl donor, contributes to initiation and progression of OSCC via epigenetic modifications like DNA hyper/hypo-methylation and histone modifications [56,57]. A study reported that tumour cells utilise methionine as a substrate to induce abnormal methylation in the tumour promoter region of the tumour suppressor gene to promote tumorigenesis and cancer development [58]. Taken together OSCC exhibits abnormally increased metabolism and absorption of certain amino acids. This is to help cancer cells survive and proliferate unhindered in the face of nutritional, genotoxic and oxidative stresses.

Not only amino acids but also purine derivatives like hypoxanthine and nucleosides like guanosine were upregulated in OSCC patients across different studies. This upregulation has been linked to increased demand for nucleotides that support nucleic acid and protein synthesis during cellular proliferation [59,60]. Hence in order to sustain increased cell growth and proliferation, tumour cells tend to replenish purine and nucleoside pool, which is correlated to the up regulated levels of purines and nucleosides [61].

In addition to amino acids and purines, many studies included in our systematic review reported down regulation of glucose and up regulation of lactic acid across three and four studies in the OSCC group respectively. This is indicative of the universal phenomena of cancer, the Warburg effect, where tumour cells increase the rate of glucose uptake (up to 20 times) and preferential production of lactic acid instead of breaking it down to CO_2_, even in the presence of oxygen [62]. Similarly, branched chain amino acids (BCAA) like valine and leucine are also upregulated across four different studies in OSCC patients. In recent years, it has become evident that various tumours uptake BCAA and oxidise them into acetyl-CoA, which then enters the TCA cycle to produce energy [63,64]. 

### 4.2. Identification of Significantly Enriched Pathways

Pathway enrichment analysis is a commonly used method to identify biological pathways that are enriched in a set of differentially regulated metabolites. This analysis has demonstrated the most influential pathways in the overall metabolic profile, which were identified as aminoacyl-tRNA biosynthesis, arginine biosynthesis, glutathione metabolism, purine metabolism, BCAA metabolism, and glycine and serine metabolism.

Firstly, Aminoacyl-tRNA synthesis (aaRSs) is known to play a fundamental role in the upkeep of proteins, primarily through loading of amino acids to tRNAs; hence, it is essential for the maintenance of cellular equilibrium and normal bodily functions [65,66]. Interestingly, in disease states such as cancer, there is evidence to suggest that Aminoacyl-tRNA synthases (ARSs) are involved in a variety of biological processes such as the regulation of inflammatory, immunological and apoptotic processes, as well as angiogenesis [67]. Recent studies have revealed their role in oncogenesis through the regulation of cell apoptosis, alteration of the tumour microenvironment and the promotion of PMN migration [68,69]. 

However, Arginine, a conditionally essential amino acid during cancer cell growth and development, is involved in a number of metabolic pathways, including the urea cycle, glycolysis and the pentose phosphate pathway [70]. It has been proposed that arginine synthesis is upregulated in cancer cells compared to normal cells, and its increased availability provides a metabolic advantage for cancer cells, allowing them to proliferate more quickly and evade apoptosis [70,71]. Several studies have found that the upregulation of arginine synthesis is linked to the increased demand for cellular components such as citrulline, nitric oxide and numerous polyamines during cancer cell proliferation causing the cancer cells to become auxotrophic to arginine, deregulating both its anabolism and catabolism [71,72,73,74]. This is because citrulline is the precursor of arginine in the urea cycle, whereas arginine is the precursor molecule for nitric oxide (NO) and polyamines [75,76]. Thus, dysregulation of metabolic pathways, including aaRS and arginine synthesis, can be attributed to tumorigenesis as well as to increased tumour growth, angiogenesis and metastasis.

In addition, to keep pace with the heightened proliferation of cancer cells and tumour growth, there is a need for more of the basic components such as proteins, nucleic acids and nucleotides, as well as additional energy [49,77]. For this metabolic pathway such as purine metabolism, BCAA metabolism, and glycine and serine metabolism are overexpressed to mitigate these needs. BCAAs include essential amino acids like leucine, valine and isoleucine. Cancer cells preferentially uptake BCAAs and either degrade them for protein synthesis or use them as a substrate in conversion of α-ketoglutarate to glutamate; they serve as an indirect source of nitrogen for nucleotide biosynthesis and become further catabolised to yield acetyl-CoA and succinyl-CoA that feed into the TCA cycle to contribute to energy production [63,78]. Moreover, it has also become evident in recent years that enzymes catalysing this degradation are overexpressed in various cancers, including pancreatic cancer and small-lung cell adenocarcinoma [78,79]. Similar to BCAAs, serine and glycine metabolism also play an important role in cancer progression by aiding in nucleotide synthesis and DNA methylation, but by acting as a source of one carbon donor [63,80]. Moreover, studies have shown that serine and glycine starvation inhibited the proliferation of cancer cells in in vitro and tumour growth in in vivo [80,81,82]. In addition to pathways related to amino acids, purines and their enzymes involved in the purine biosynthesis pathway are increased in tumours because purine nucleotides are critical for tumour cell growth [61]. This is because the three metabolites involved in this metabolic pathway, hypoxanthine, xanthine, and uric acid, are elevated by cancer cells to promote nucleic acid production, which substantiates the accelerated cancer cell growth and proliferation [83]. Altogether, dysregulation of BCAA and non-essential amino acid metabolism as well as purine metabolism are essential for the upregulated synthesis of essential cellular components and energy.

In the end, for cancer progression, the ability to survive and resist treatment are just as essential as increased growth and development. Glutathione, which is an antioxidant produced by our body, is responsible for this, taking part in various cellular activities such as detoxification and keeping cells in a balanced reduction-oxidation state [84]. Numerous studies on various malignancies, however, have found that glutathione metabolism is connected to carcinogenesis and progression by involving in the regulation of autophagy in cancer cells, which is important for the maintenance of cancer cell survival [85,86]. In addition, a rise in glutathione expression promotes antioxidant mechanisms and several oncogenic pathways, such as the PI3K/Akt/mTOR and NF-κB pathways, as well as modulating the apoptotic mechanisms in cancer cells, making them more resistant to oxidative stress and apoptosis [87]. Thus, glutathione metabolism is significant for the cancer’s proliferation, survival and response to the treatment.

### 4.3. Limitations

This is, to the best of our knowledge, the first systematic review focusing on the application of global metabolomics across various sample types and analytical methods to identify several metabolites and their dysregulated pathways, which can distinguish OSCC patients from healthy people, consistent with other cancer metabolomics-based systematic reviews. However, its shortcomings primarily attributable to the methodology of the included studies should be acknowledged as well.

First, most of the studies included scored poorly in factors such as sample size justification and randomisation. Both can be attributed to the small sample sizes used in many studies. Small sample size may also result in the diminished power of the study and the lack of applicability of the results. Second, the majority of the studies considered did not include raw data, but instead only selectively reported data, resulting in reporting bias and the overlooking of potential metabolites. Even if raw data is available, there is little information on how it is handled and filtered.

Third, results were not reproducible due to the opaque methodology and a lack of validation of the reported metabolites. Next, many of the studies did not adequately address the confounding risk factors associated with OSCC, which makes it difficult to understand how these risk factors might potentially modulate the expression of some metabolites and their pathways. Finally, despite the stricter inclusion criteria, there was heterogeneity in the sample type, OSCC lesion location and stage, sample collection, processing and the analytical method employed. These factors significantly impact the results and may be one of the leading factors for the inconsistencies in the metabolite concentrations between studies.

## 5. Conclusions

For many years, early detection of OSCC has been a major challenge. However, this could change with the emergence of the developing field of metabolomics. Despite several limitations, this systematic review identified several metabolite biomarkers and their associated dysregulated pathways that distinguished OSCC from healthy controls. L-glutamic acid, L-methionine, hypoxanthine and Warburg effect metabolites (glucose and lactic acid) were particularly promising. 

Based on our systematic review and the challenges we encountered, we foresee the need for improvement in validating the identified features in larger cohorts and standardizing the recommendations for sample preparation and data processing. This would allow the future researchers to confirm the findings of previous studies and make it easier to interpret and reproduce results and to develop clinical applications for metabolomics. Finally, creating cost-effective, fast-paced analytical platforms and integrating them with AI and ML can make the metabolomic approach a more reliable and useful tool for the clinical diagnosis and treatment of OSCC.

## Figures and Tables

**Table 1:** Detailed characteristics of the included studies.

Author/Year	Country	Cases/Controls	Age (Range)	Sex (Male/Female)	Cancer Staging (I/II/III/IV)	Inclusion/Exclusion Criteria for Cases	Sample Type	Sample Storage	Sample Preparation (Yes/No)	Analytical Methods
De Sa Alves., (2021) [37]	Brazil	27 OSCC/41 Healthy controls	OSCC: 57 ± 13.87 Healthy Controls: 57.34 ± 11.66 [mean ± SD] (28–88)	OSCC:19/8 Healthy controls: 21/20	4/4/6/13	IC: Patients aged 18 & above with confirmed diagnosis of OSCC. EC: Patients diagnosed with other cancers/have undergone prior treatment with surgery, chemo/radio therapy	Saliva	−80 °C	Yes	GC-MS
Enomoto et al., (2018) [38]	Japan	48 OSCC/29 other oral diseases	OSCC: 66.3 Other oral diseases: 60.3 [mean]	OSCC: 25/23 Other oral diseases: 15/14	9/10/11/18	IC: Confirmed OSCC EC: History of malignant tumour, metabolic disease, or endocrine disease	Serum	−80 °C	Yes	GC-MS
Ishikawa et al., (2016) [35]	Japan	24 OC/44 Healthy controls	OC: 72 (23–94) Healthy controls: 68 (21–90) [median]	OC: 14/10 Healthy controls: 16/28	5/6/8/5	Not reported	Saliva, Tissue	−80 °C	Yes	CE-TOFMS
Ishikawa et al., (2017) [36]	Japan	22 OSCC/44 Healthy controls	OSCC: 72 (23–94) Healthy controls: 68 (21–90) [median]	OSCC: 12/10 Healthy controls: 16/28	3/6/8/5	IC: None of the OSCC patients received prior chemo/radio treatment. EC: History of malignancies or autoimmune disorders	Saliva	−80 °C	Yes	CE-TOFMS
Ishikawa et al., (2018) [39]	Japan	6 OSCC, 10 OED/32 PSOML	OSCC: 63.5 (49–83) OED: 69 (57–81) PSOML: 62.5 (21–86) [median]	OSCC: 6/0 OED: 6/4 PSOML: 21/11	NR	IC: Pathologically confirmed OSCC, OED and OELP. EC: Prior chemo/radio therapy	Saliva	−80 °C	Yes	CE-TOFMS
Ishikawa et al., (2019) [40]	Japan	34 OSCC/26 OLP	OSCC: 70.5 (29–87) OLP: 67.5 (34–98) [median]	OSCC: 20/14 OLP: 5/21	14/9/2/9	IC: Pathologically confirmed OSCC, OLP. EC: Prior chemo/radio therapy	Saliva	−80 °C	Yes	CE-TOFMS
Li et al., (2022) [23]	China	72 OSCC,75 OELP/47 Healthy controls	OSCC: 66 ± 12 OELP: 61 ± 7 Healthy controls: 65 ± 9 [mean ± SD]	OSCC: 35/37 OELP: 38/37 Healthy controls: 23/24	17/21/19/14 Unknown: 1	IC: Pathologically confirmed OSCC, OELP confirmed as per WHO diagnostic criteria of lichen planus. EC: No released/refractory OSCC/OELP, free from chronic systemic diseases	Serum	−80 °C	Yes	UHPLC-Q-Orbitrap
Song et al., (2019) [22]	China	125 OSCC, 124 PML/124 Healthy controls	OSCC: 35–65 PML: 35–65 Healthy controls: 30–60	OSCC: 65/60 PML: 64/60 Healthy controls: 64/60	29/40/23/33	IC: Histologically confirmed OSCC, PML. EC: Prior chemo/radio therapy	Saliva	−80 °C	Yes	CPSI-MS coupled with ML
Sridharan et al., (2019) [41]	India	22 OSCC, 21 OLK/18 Healthy controls	OSCC: 43 OLK: 48 Healthy controls: 32 [median]	OSCC: 81.9%/18.1% OLK: 90.5%/9.5% Healthy controls: 66.7%/33.3%	NR	IC: OSCC: clinical and histopathological confirmed OSCC; OLK: clinically diagnosed OLK. EC: history of systemic illness and medications; history of therapy for OLK and OSCC and with recurrent oral lesions.	Saliva	−80 °C	Yes	UPLC-QTOFMS
Syed et al., (2016) [42]	Pakistan	21 OSCC, 15 OSF/15 Healthy controls	NR	NR	NR	IC: Clinically confirmed OSCC and OSF. EC: Prior therapy and in either remission or relapse stage.	Tissue	−80 °C	Yes	GC-MS
Tsai et al., (2020) [43]	Taiwan	110 OSCC (37 normal tissue, 36 tumour tissue, 44 plasma, 98 urine)	52.4 (28–79) [median]	NR	NR	IC: Oral cavity cancers EC: Any other tumours including oropharyngeal cancers	Tumour tissue, plasma & urine	−80 °C	Yes	NMR
Yang et al., (2020) [44]	China	8 OSCC/8 Healthy controls	NR	NR	NR	Not Reported	Tumour tissue	−80 °C	Yes	GC-MS
Yang et al., (2021) [20]	China	578 OSCC/241 Healthy controls	NR	NR	NR	IC: Histopathological confirmed OSCC. EC: Not reported.	Serum	−80 °C	Yes	CPSI-MS

OSCC: Oral squamous cell carcinoma, OELP: Oral erosive leukoplakia, PML: Premalignant lesion, OLK: Oral leukoplakia, OSF: Oral sub-mucous fibrosis, IC: Inclusion criteria, EC: Exclusion criteria, GC: Gas chromatography, LC: Liquid chromatography, MS: Mass spectrometer, CE: Capillary electrophoresis, NMR: Nuclear magnetic resonance, CPSI: Conductive polymer spray ionisation, UHPLC: Ultra-high performance liquid chromatography, UPLC-QTOF-MS: Ultra Performance Liquid Chromatography coupled to a hybrid quadrupole orthogonal time of flight mass spectrometer, TOF: Time of flight, ML: Machine learning, CE-TOF/MS: Capillary electrophoresis time-of-flight mass spectrometry, SD: Standard deviation, NR: Not reported in the original publication.

## Data Availability

The data used in this study can be obtained from the corresponding author upon request. Being a systematic review, the data are contained in an Excel spreadsheet and are already displayed in most of the tables and figures in the manuscript.

## Supplementary Materials

The following supporting information can be downloaded at: https://www.mdpi.com/article/10.3390/metabo13080890/s1; Table S1: Detailed search strategy across the databases; Table S2: Risk of Bias and Quality Assessment; Table S3: Metabolite subgrouping into superclass and subclass.
