# Supplementary material for: Evaluation of Metabolomics as Diagnostic Targets in Oral Squamous Cell Carcinoma: A Systematic Review
Source: Metabolites. 2023 Jul 27;13(8):890. doi: 10.3390/metabo13080890 (PMC10456490; doi:10.3390/metabo13080890)
Supplement: Supplementary file 1 [file metabolites-13-00890-s001.zip › metabolites-2509192-supplementary.pdf]

SUPPLEMENTARY FILE

Title: Evaluation of metabolomics as diagnostic targets in oral squamous cell carcinoma: a systematic review.

Authors: Susanth Alapati[1], Giulio Fortuna[2], Gordon Ramage[1], and Christopher Delaney[1]*

*Correspondence: christopher.delaney@glasgow.ac.uk

[1]Oral Sciences Research Group, Glasgow Dental School, School of Medicine, Dentistry and Nursing, University of Glasgow, 378 Sauchiehall Street, Glasgow, United Kingdom, G2 3JZ

[2]Department of Oral Medicine, Glasgow Dental School, School of Medicine, Dentistry and Nursing, University of Glasgow, 378 Sauchiehall Street, Glasgow, United Kingdom, G2 3JZ

Supplementary Table S1: Detailed search strategy across the databases.

Database	No.	Search Statement
**Medline (Ovid)**	1	(oral or mouth or "oral cavity" or oropharyn* or tongue or "base of tongue" or "floor of mouth" or palat* or lingual or buccal or lip or labial or "oral mucosa*" or retromolar or cheek or gingiva* or intra-oral or vermillion).mp.
	2	(cancer* or tumor* or tumour* or malignant* or neoplasm* or carcinoma* or squamous or squamous cell* or OSCC or soft tissue or HPV-positive or HPV-negative or HPV+ve or HPV-ve).mp.
	3	(metabolomic* OR metabonomic* OR "metabolic profiling" OR metabolome OR "metabolite marker" OR "metabolite biomarker").mp.
	4	1 AND 2 AND 3
Database	No.	Search Statement
**Embase (Ovid)**	1	(oral or mouth or "oral cavity" or oropharyn* or tongue or "base of tongue" or "floor of mouth" or palat* or lingual or buccal or lip or labial or “oral mucosa*” or retromolar or cheek or gingiva* or "intra-oral" or vermillion).mp.
	2	(cancer* or tumor* or tumour* or malignant* or neoplasm* or carcinoma* or squamous or squamous cell* or OSCC or soft tissue or HPV-positive or HPV-negative or HPV+ve or HPV-ve).mp.
	3	(metabolomic* OR metabonomic* OR 'metabolic profiling' OR metabolome OR 'metabolite marker' OR 'metabolite biomarker').mp
	4	1 and 2 and 3
Database	No.	Search Statement
**Cochrane**	1	(oral or mouth or "oral cavity" or oropharyn* or tongue or "base of tongue" or "floor of mouth" or palat* or lingual or buccal or lip or labial or “oral mucosa*” or retromolar or cheek or gingiva* or "intra-oral" or vermillion)
	2	(cancer* or tumor* or tumour* or malignant* or neoplasm* or carcinoma* or squamous or squamous cell* or OSCC or soft tissue or HPV-positive or HPV-negative)
	3	(metabolomic* OR metabonomic* OR "metabolic profiling" OR metabolome OR "metabolite marker" OR "metabolite biomarker")
	4	1 and 2 and 3

Database	No.	Search Statement
**CINAHL**	1	(oral or mouth or "oral cavity" or oropharyn* or tongue or "base of tongue" or "floor of mouth" or palat* or lingual or buccal or lip or labial or “oral mucosa*” or retromolar or cheek or gingiva* or "intra-oral" or vermillion) ‘All text’
	AND 2	(cancer* or tumor* or tumour* or malignant* or neoplasm* or carcinoma* or squamous or squamous cell* or OSCC or soft tissue or HPV-positive or HPV-negative or HPV+ve or HPV-ve) ‘All text’
	AND 3	(metabolomic* OR metabonomic* OR "metabolic profiling" OR metabolome OR "metabolite marker" OR "metabolite biomarker") ‘All text’
	AND 4	1 AND 2 AND 3

Database	Search Statement
**Web of Science Core Collection**	TS=(oral or mouth or "oral cavity" or oropharyn* or tongue or "base of tongue" or "floor of mouth" or palat* or lingual or buccal or lip or labial or “oral mucosa*” or retromolar or cheek or gingiva* or "intra-oral" or vermillion) AND<br>TS=(cancer* or tumor* or tumour* or malignant* or neoplasm* or carcinoma* or squamous or squamous cell* or OSCC or soft tissue or HPV-positive or HPV-negative or HPV+ve or HPV-ve) AND<br>TS=(metabolomic* OR metabonomic* OR "metabolic profiling" OR metabolome OR "metabolite marker" OR "metabolite biomarker")

Database	Search Statement
**ProQuest**	(TI,AB,IF(oral) OR TI,AB,IF(mouth) OR TI,AB,IF("oral cavity") OR TI,AB,IF(oropharyn*) OR TI,AB,IF(tongue) OR TI,AB,IF("base of tongue") OR TI,AB,IF("floor of mouth") OR TI,AB,IF(palat*) OR TI,AB,IF(lingual) OR TI,AB,IF(buccal) OR TI,AB,IF(lip) OR TI,AB,IF(labial) OR TI,AB,IF("oral mucosa*") OR TI,AB,IF(retromolar) OR TI,AB,IF(cheek) OR TI,AB,IF(gingiva*) OR TI,AB,IF(intra-oral) OR TI,AB,IF(vermillion)) AND (TI,AB,IF(cancer*) OR TI,AB,IF(tumor*) OR TI,AB,IF(tumour*) OR TI,AB,IF(malignant*) OR TI,AB,IF(neoplasm*) OR TI,AB,IF(carcinoma*) OR TI,AB,IF(squamous) OR TI,AB,IF("squamous cell*") OR TI,AB,IF(OSCC) OR TI,AB,IF("soft tissue") OR TI,AB,IF(HPV-positive) OR TI,AB,IF(HPV-negative) OR TI,AB,IF(HPV+ve) OR TI,AB,IF(HPV-ve)) AND (TI,AB,IF(metabolomic*) OR TI,AB,IF(metabonomic*) OR TI,AB,IF("metabolic profiling") OR TI,AB,IF(metabolome) OR TI,AB,IF("metabolite marker") OR TI,AB,IF("metabolite biomarker"))

Table S2

	Ishikawa et al., (2016) [35]	Syed et al., (2016) [42]	Ishikawa et al., (2017) [36]	Enomoto et al.,(2018) [38]	Ishikawa et al., (2018) [39]	Ishikawa et al., (2019) [40]	Sridharan et al, (2019) [41]	De Sa Alves et al., (2021) [37]	Song et al., (2019) [22]	Tsai et al., (2020) [43]	Yang et al., (2020) [44]	Yang et al., (2021) [20]	Li et al., (2022) [23]
**Clearly stated research question and objectives**	Yes	Yes	Yes	Yes	Yes	Yes	Yes	Yes	Yes	Yes	Yes	Yes	Yes
**Valid inclusion and exclusion criteria**	Not clear	Yes	Not clear	Yes	Yes	Yes	Yes	Yes	Yes	Yes	Yes	No	Yes
**Spectrum of patients representative of the disease of interest**	Yes	Yes	Yes	Yes	Yes	Yes	Yes	Yes	Yes	Yes	Yes	Yes	Yes
**Sample size justification**	No	No	No	No	No	No	Yes	No	No	No	No	No	No
**Type of sample fully described**	Yes	Yes	Yes	Not clear	Yes	Yes	Yes	Not clear	Yes	Yes	Not clear	Yes	Not clear
**Description of sample collection, handling and storage**	Not clear	Yes	Yes	Yes	Yes	Yes	Yes	Yes	Yes	Yes	Not clear	Yes	Not clear
**Confounding factors like smoking, diet interferences addressed**	No	No	No	No	No	No	No	Not clear	Not clear	Not clear	No	No	No
**Randomisation of samples**	No	No	No	Yes	No	No	No	No	No	No	No	No	No
**Description of pre-analytical processing of sample**	Yes	Yes	Yes	Yes	Yes	Not clear	Yes	Yes	Yes	Yes	Not clear	Yes	Yes
**Analytical methods clearly described**	Yes	Yes	Yes	Yes	Yes	Yes	No	Yes	Yes	Yes	Not clear	Yes	Yes
**Clearly defined measures of risk/exposure**	Yes	No	Yes	Yes	Yes	Yes	Yes	Not clear	Yes	Yes	Yes	Yes	Yes
**Comprehensive details on statistical analysis**	Yes	No	Yes	Yes	Yes	Yes	Not clear	Yes	Yes	Yes	Yes	Yes	Yes

R-Script for differentiation of upregulated and downregulated metabolites.

```
#library(dbplyr)
#library(dplyr)
#library(tidyverse)
#Com_FoldChange <- read.csv("filename.csv")
#unique(Com_FoldChange$Comparison)
#Com_FoldChange<-Com_FoldChange %>% mutate(Comparison_order
=Com_FoldChange$`Comparison order`) %>% separate(.,'Comparison order', into = c("Positive",
"Negative"), sep = "_")
#Com_FoldChange<-Com_FoldChange %>% mutate(Comparison_order
=Com_FoldChange$`Comparison.order`) %>% separate(.,'Comparison.order', into = c("Positive",
"Negative"), sep = "_")
#View(Com_FoldChange)
#Com_FoldChange$log2_FoldChange<-log2(as.numeric(Com_FoldChange$`Fold Change`))
#Com_FoldChange$Metaboanalyst_name<-Com_FoldChange$`Metaboanalyst matched name`
#View(Com_FoldChange)
#Com_FoldChange_OSCC<-Com_FoldChange %>% filter(Positive =="OSCC")
#View(Com_FoldChange_OSCC)
#OSCC_UPregulated<- Com_FoldChange_OSCC %>% filter(log2_FoldChange >0)
#Studys_OSCC<-unique(Com_FoldChange_OSCC$Study)
#Com_FoldChange_OSCC<-Com_FoldChange %>% filter(Positive %in% c("OSCC")) %>%
#filter(Negative=="HC")
#View(Com_FoldChange_OSCC)
#OSCC_UPregulated <- Com_FoldChange_OSCC %>% filter(log2_FoldChange >0) %>%
#group_by(Metaboanalyst_name) %>%
#summarise(n = n())
#OSCC_DOWNregulated <- Com_FoldChange_OSCC %>% filter(log2_FoldChange <0) %>%
#group_by(Metaboanalyst_name) %>%
#summarise(n = n())
```

Supplementary Table S3: Metabolite subgrouping into superclass and subclass.

**Organic acid derivatives**	**119**
Amino acids, peptides, and analogues	89
Others Carboxylic acid derivatives	15
Hydroxy acids and derivatives	4
Keto acids and derivatives	3
Phosphate esters	3
Carboximidic acids and derivatives	3
Sulfinic acids and derivatives	2
**Lipids**	**94**
Fatty acids and Fatty acyls	48
Other lipid derivatives	14
Glycerophospholipids	12
Glycerols	8
Bile acids	6
Steroid esters	6
**Organoheterocyclic compounds**	**46**
Purines and purine derivatives	14
Pyrimidines and pyrimidine derivatives	13
Other olines, lignans and ureas	9
Pyridines and derivatives	4
Piperidines	2
Azoles	2
Indoles and derivatives	2
**Organic oxygen compounds**	**49**
Alcohols and polyols	6
Carbohydrates	43
**Benzene and Benzene derivatives**	**11**
Benzene and Benzene derivatives	8
Phenol and Phenol derivatives	3
**Organic nitrogen compounds**	**9**
Quaternary ammonium salts	5
Amines	3
Aminoxides	1
**Nucleosides**	**3**
Purine and Pyrimidine Nucleosides	2
5'-deoxyribonucleosides	1
**Homogeneous non-metal compounds**	**2**
Non-metal phosphates	2
**Polyketides**	**4**
Phenylpropanoids	3
Flavonoids	1
